# Comprehensive assessment of aortic flow before and after aortic valve replacement in an *ex vivo* porcine model with four-dimensional flow magnetic resonance imaging

**DOI:** 10.1093/icvts/ivaf087

**Published:** 2025-04-09

**Authors:** Hiroyuki Saisho, Maren Friederike Balks, Michael Scharfschwerdt, Tim Schaller, Najla Sadat, Anas Aboud, Stephan Ensminger, Alex Frydrychowicz, Buntaro Fujita, Thekla Helene Oechtering

**Affiliations:** Department of Cardiac and Thoracic Vascular Surgery, University Hospital Schleswig-Holstein, Lübeck, Germany; Department of Radiology and Nuclear Medicine, University Hospital Schleswig-Holstein, Lübeck, Germany; Section of Pediatric Radiology, Department of Diagnostic and Interventional Radiology and Nuclear Medicine, University Medical Center Hamburg-Eppendorf, Hamburg, Germany; Department of Cardiac and Thoracic Vascular Surgery, University Hospital Schleswig-Holstein, Lübeck, Germany; Department of Cardiac and Thoracic Vascular Surgery, University Hospital Schleswig-Holstein, Lübeck, Germany; Department of Cardiac and Thoracic Vascular Surgery, University Hospital Schleswig-Holstein, Lübeck, Germany; Department of Cardiac and Thoracic Vascular Surgery, University Hospital Schleswig-Holstein, Lübeck, Germany; Department of Cardiac and Thoracic Vascular Surgery, University Hospital Schleswig-Holstein, Lübeck, Germany; Department of Radiology and Nuclear Medicine, University Hospital Schleswig-Holstein, Lübeck, Germany; Department of Cardiac and Thoracic Vascular Surgery, University Hospital Schleswig-Holstein, Lübeck, Germany; Department of Radiology and Nuclear Medicine, University Hospital Schleswig-Holstein, Lübeck, Germany; Department of Radiology, University of Wisconsin, Madison, WI, USA

**Keywords:** *ex vivo* model, AVR, 4D flow MRI, Ozaki

## Abstract

**OBJECTIVES:**

Aortic valve replacement (AVR) has shown to induce secondary flow patterns deviating from main flow. It is impossible to analyse the impact of surgical access and different AVR techniques under standardized conditions in patients or silicone models. Therefore, we developed an *ex vivo* swine model to analyse the impact of surgical access and to compare flow patterns after different AVR techniques within the ascending aorta.

**METHODS:**

Porcine aortas (*n* = 6) were anastomosed to a custom-made piston pump. The pulse duplicator perfused the aortas with a blood-mimicking fluid at 2.5 l/min and 64 bpm. 4D flow magnetic resonance imaging of each aorta was acquired prior to surgery (NAV, *n* = 6), after sham surgery (aortotomy and closure thereof without valve replacement, NAV-A, *n* = 6) and after Ozaki procedure (AVneo, *n* = 2), biological valve (BV, *n* = 2) or mechanical valve (MV, *n* = 2). Secondary flow patterns and peak velocity were analysed with GTFlow (GyroTools, Switzerland).

**RESULTS:**

Sham surgery alone induced secondary flow patterns in the ascending aorta in all specimens. After AVR, more secondary flow patterns were observed distal to BV compared to AVneo or MV. Three flow patterns developed after BV, two after AVneo and one after MV. In addition, peak velocity within the aortic sinuses of Valsalva increased after all AVR procedures, most strikingly after BV (NAV = 75 ± 22 cm/s, NAV-A = 79 ± 29 cm/s, AVneo = 115 ± 36 cm/s, BV = 142 ± 21 cm/s, MV = 107 ± 4 cm/s; mean±standard deviation).

**CONCLUSIONS:**

We successfully established an *ex vivo* model suggesting that flow alterations not only depend on the type of AVR but are associated with surgical access. The strongest secondary flow patterns developed after BV followed by AVneo and MV.

## Graphical Abstract

**Figure:**
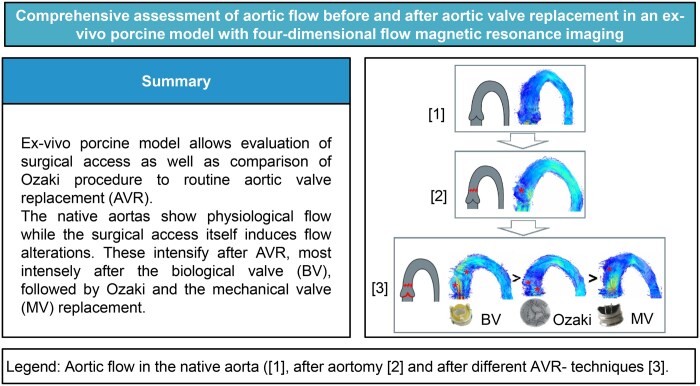

## INTRODUCTION

There are various types of aortic valve replacement (AVR) available. Mechanical valves (MVs) offer a low risk of reoperation due to very long durability but require lifelong effective anticoagulation. In contrast, biological valves (BVs) do not require anticoagulation but are subject to degeneration and therefore have limited durability. Another option is the aortic valve neocuspidization technique after Ozaki [1], where the patient’s own pericardium is used to replace the aortic valve cusps. Three new leaflets are trimmed from the autologous pericardium of the patient. The annular margins of the leaflets are then sewn into the aortic annulus to construct the new valve without the need for lifelong anticoagulation.

4D flow magnetic resonance imaging (MRI) examinations in patients after AVR have shown the persistence of secondary flow patterns and eccentric flow in the ascending aorta after BV replacement compared to healthy volunteers [2, 3]. Postoperative flow might be influenced by individual aortic anatomy. Therefore, we previously examined different AVR techniques in the same aortic geometry of a silicone model with near-physiological conditions. Here, we detected more secondary flow patterns deviating from normal main flow after BV replacement in contrast to MV replacement independent of the geometry of the aorta [4]. Secondary flow patterns are associated with changes in wall shear stress (WSS), which can trigger a cascade of pathological responses in the endothelial cells that can lead to atherosclerosis, changes in the aortic wall integrity, and aneurysm formation. Secondary flow patterns also influence cardiac and valve function [5, 6].

The impact of surgical procedures like the Ozaki procedure cannot be tested in silicone models as the required suturing of the pericardial leaflets cannot be performed. Moreover, the influence of surgical access (aortotomy) cannot be evaluated. Closing of the aortotomy may lead to increased local vessel stiffness and altered geometry from the suture. Therefore, the purpose of this study was to establish an *ex vivo* swine model to evaluate the impact of the surgical access site and different valve replacements under standardized, (near-)physiological conditions.

## MATERIALS AND METHODS

### Experimental setup and surgical technique

Fresh porcine hearts including the thoracic aorta were retrieved from a local butcher and used for this *ex vivo* model (*n* = 6). Consent was therefore not required. The aortas were only used if sizing allowed for the implantation of a valve with an outer diameter of 21 mm. All AVRs were performed by the same experienced cardiothoracic surgeons.

**Figure 1:**
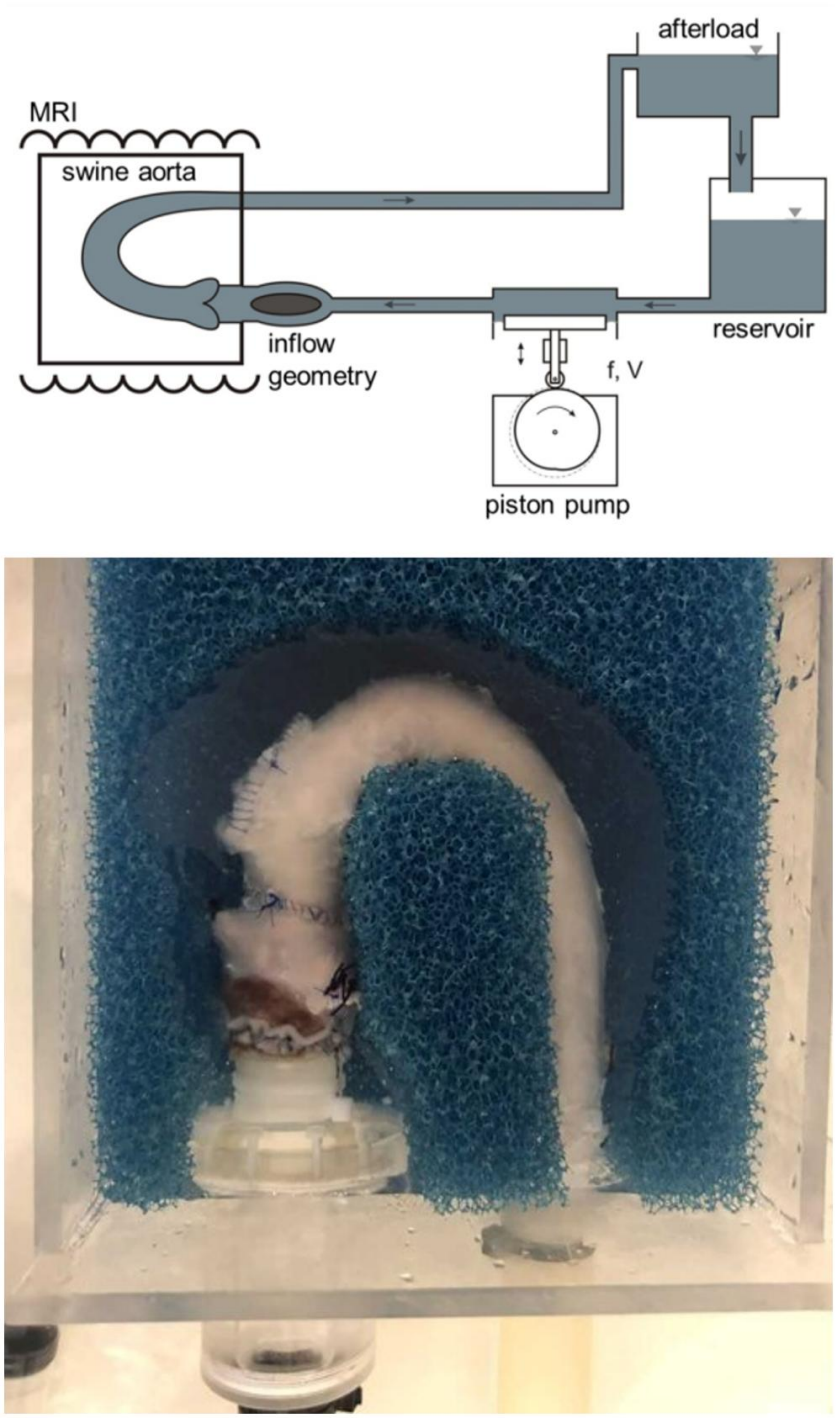
Setup of *ex vivo* model with swine aorta. Top: Aorta anastomosed to a custom-made pump with a conic inflow geometry. The pump was positioned at the end of the MRI examination table. The afterload-controlled pump pumps blood-mimicking fluid with an average flow of 2.2 l/min. Bottom: The aorta (hereafter Ozaki) in the setup for 4D flow MRI. During the MRI examination, the aorta was positioned in a dedicated acrylic glass box with cutout foam material in keep the aorta in position

The left ventricular outflow tract (LVOT) was dissected. All branches of the aorta, including the coronary arteries, supra-aortic vessels and intercostal arteries, were ligated. Short segments of 28 mm Dacron prostheses were sewn to the LVOT and the descending aorta. The Dacron prostheses were connected to an MRI-compatible, custom-made mock circulation loop which contained an in-house-made pulse duplicator (Fig. 1). The setup had a conic inflow geometry to allow for a more homogenous velocity profile compared to a simple cylindrical inflow geometry [7]. In comparison to the human aorta, the porcine aorta has a relatively short ascending aorta prior to the supra-aortic vessels. We therefore positioned the aorta in a dedicated acrylic glass box with cutout foam material to maintain a position and angulation that was similar to a human aorta with a relatively longer ascending part (Fig. 1). The box was filled with water to provide signal for the MRI acquisition and minimize artefacts at a fluid-air border.

The loop was perfused with blood-mimicking fluid containing 36.6% glycerin in physiological saline with a viscosity similar to blood [8]. During the measurement, the in-house-made piston pump perfused the setup with a physiological flow profile at 2.5 l/min and 64 bpm, respectively. The ratio of systole/diastole was 35%/65%. The model was connected to a homebuilt afterload of 1.09 m water column (i.e. 80 mmHg) to allow for realistic diastolic pressure.

**Figure 2:**
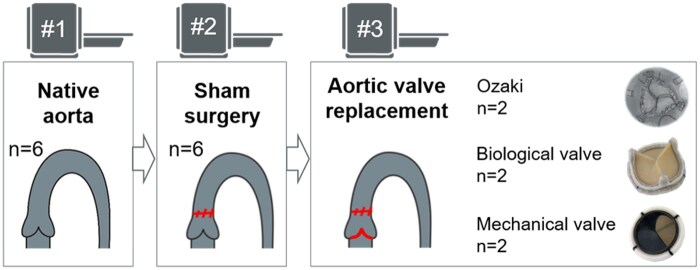
Each porcine aorta was examined three times: (i) with the native aortic valve, (ii) after aortotomy (sham surgery) and (iii) after aortic valve replacement (Ozaki/biological valve/mechanical valve)

Each aorta was analysed three times: In the first step, the six aortas with their native aortic valve were examined with 4D flow MRI to get baseline flow profile (NAV, *n* = 6). Secondly, a transverse aortotomy was performed in each of the aortas (NAV-A, *n* = 6). Each aorta was incised cranial to the origin of the right coronary artery in the ascending aorta. The cut was extended towards the non-coronary cusp on one side and obliquely parallel to the origin of the left coronary artery on the other. Closure was performed with 4–0 polypropylene using a double-layer technique with a running horizontal mattress suture followed by a simple running (over-and-over) suture. In a third step, the native aortic valve was surgically replaced by one of the following three substitutes which are clinically used in our institution: the Ozaki neocuspidized valve (‘AVneo’; *n* = 2), a BV (PERIMOUNT Magna Ease 21 mm; Edwards Lifesciences LLC, Irvine, CA, USA; *n* = 2), or a MV (St Jude Medical Masters HP^TM^ mechanical heart valve 21 mm, St Jude Medical Inc., St Paul, MN, USA; *n* = 2; Fig. 2). AVneo was performed according to the protocol by Ozaki *et al.* [1] using porcine pericardium. The pericardium was treated with 0.6% glutaraldehyde and washed with saline. Implantation of the two prosthetic valves was performed in the usual manner using 2-0 polyester sutures with felt pledgets that were positioned supra-annularly (non-everting suture technique). The bileaflet MVs were implanted not to interfere coronary flow and were adjusted so that one of its hinges was positioned between the ostia of both coronary arteries [9].

### MRI scan and processing of MR data

A 3D, three-directionally velocity-sensitive, time-resolved phase contrast sequence (4D flow MRI) was employed at 3 Tesla (Philips Ingenia Omega, Philips Healthcare, Netherlands) using a 20-channel body surface coil. The sequence was retrospectively ECG-gated with a mock ECG signal that was generated by the pump synchronous to its pump rate.

Typical imaging parameters included isotropic voxels (resolution = 2.45 × 2.5 × 2.5 mm³, reconstructed to 1.24 × 1.24 × 1.45 mm³) and a field-of-view of 168 × 178 mm with 25–35 slices in oblique sagittal orientation, carefully adapted to each aorta. Further scan parameters were repetition time = 4.0 ms, echo time = 2.8 ms, Cartesian encoding, SENSE acceleration factor = 2.2, no respiratory gating, and a total acquisition time of 4 min. Data were reconstructed at the MRI system and transferred to an offline computer for postprocessing with GTFlow (v3.2.13, GyroTools, Zurich, Switzerland). Reconstruction and postprocessing included correction for errors due to background phase offsets, Maxwell terms and eddy currents.

**Figure 3:**
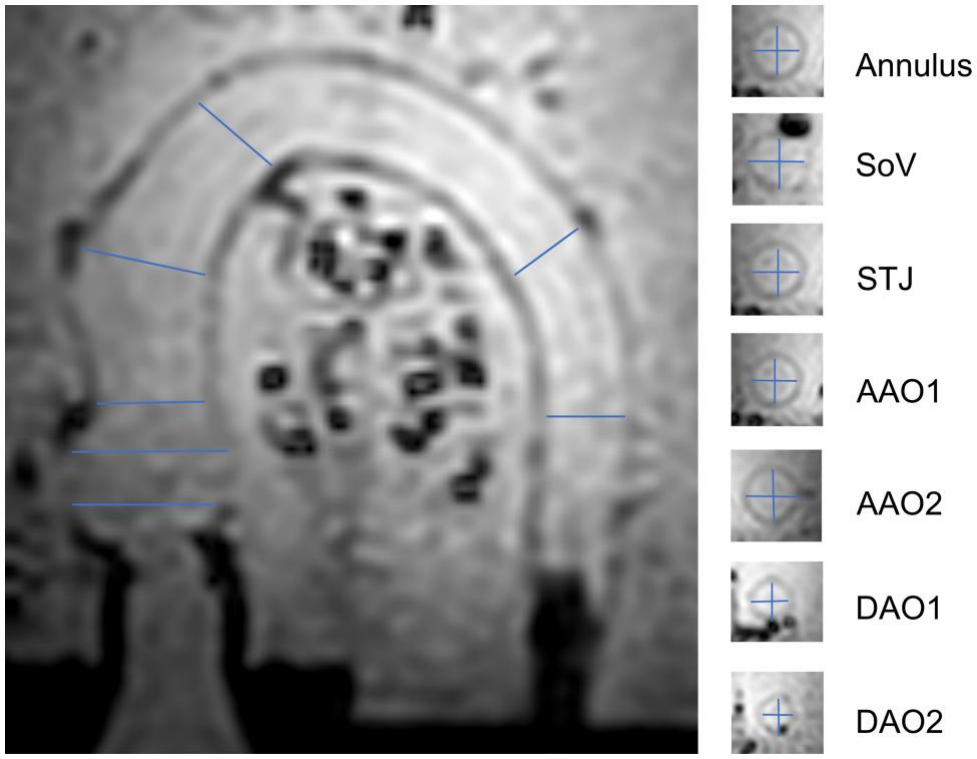
4D flow MRI-derived magnitude image, an example of measurement at the annulus, the sinus of Valsalva (SoV), the sinutubular junction (STJ), the ascending aorta (AAO1, AAO2) and the descending aorta (DAO1, DAO2)

Diameter of the porcine aortas was measured at standardized planes in their native state under examination conditions in systole on the 4D flow magnitude image (Fig. 3).

### Analysis of 4D flow MRI data

**Figure 4:**
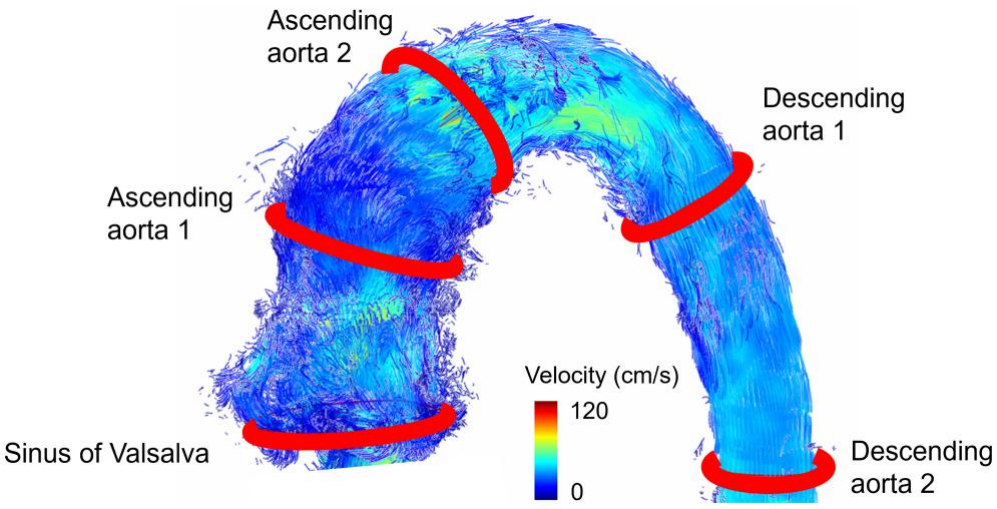
Defined 2D contours in the aorta for analysis of flow characteristics and emitter planes for particle traces (*n* = 5): at the level of the sinuses of Valsalva, two contours in the ascending aorta and two contours in the descending aorta

Five 2D contours were manually placed at anatomical landmarks in the aorta orthogonal to the vessel (Fig. 4) using 4D flow images. All contours were adjusted to the aorta for each time frame. Aortic flow was visualized by particle traces emitted from the contours and arriving at the contours (‘backward and forward’ setting in GTFlow). Particle traces were colour-coded according to the velocity. We distinguished between helices and vortices. In a helix, the blood moves spirally in main flow direction, in a vortex it is recirculating and deviating from main flow direction [10, 11]. Helical flow, encompassing the complete aortic cross section was considered a physiological, primary flow pattern [10]. Helices with a smaller diameter as well as vortices were defined as secondary flow pattern. Secondary flow patterns deviating from main flow were graded on a Likert scale in a four-step approach [11] using 2D and 4D visualization according to their diameter in relation to the vessel cross section. They were graded as small (grade I), medium (grade II) and large (grade III) (Supplementary Material, Fig. S1). Therefore, the physiological sinus vortices distal to the aortic valve reach high into the ascending aorta. Due to these anatomical peculiarities of the porcine aorta quantitative parameters were measured at the aortic bulb. Quantitative parameters were measured at the sinuses of Valsalva and included stroke volume (ml) and peak maximal velocity (cm/s, maximal velocity of one voxel of the contour). Results are given as mean ± standard deviation. Statistical analysis was descriptive due to the small group size.

## RESULTS

We successfully examined six aortas in their native state, after sham surgery and after AVR. Measurements of the aortas were 25.3 ± 3.3 mm at the annulus, 30.3 ± 2.8 mm at the sinuses of Valsalva, 21.5 ± 0.4 mm at the sinotubular junction, 24.9 ± 2.1 mm at mid ascending aorta, 18.8 ± 2.1 mm at the distal ascending aorta, 15.8 ± 0.9 mm at the proximal descending aorta and 15.5 ± 0.8 mm at the mid descending aorta.

Physiological systolic flow without any secondary flow patterns was observed in all but one native aorta. One specimen (BV2) had a subvalvular stenosis at the anastomosis between LVOT and prosthesis characterized by increased velocity in the aortic root and ascending aorta, caused by a tight anastomosis between the outflow tract and the prosthesis that connects the aorta to the mock circulation. This was only visualized after the experiment and could not be changed.

**Figure 5:**
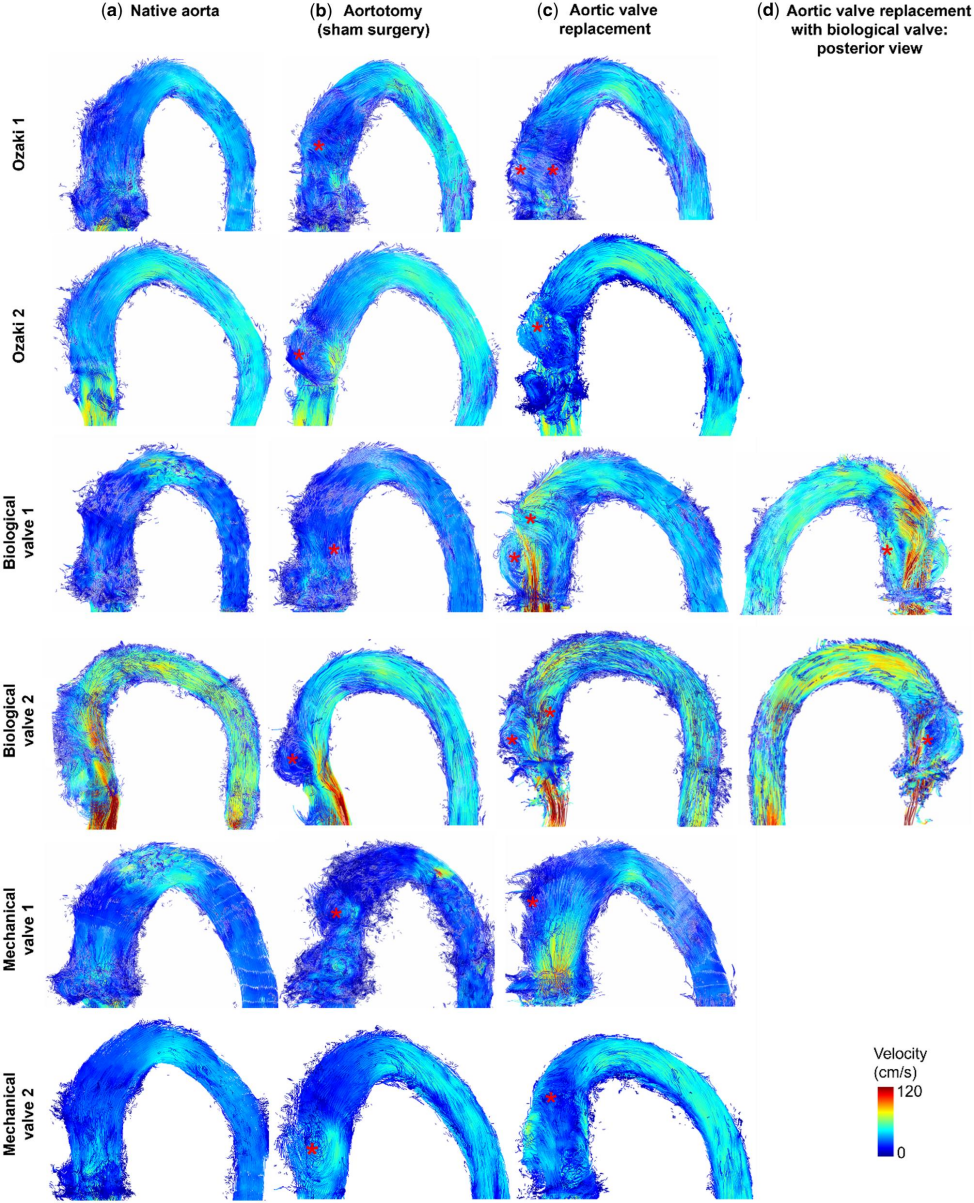
Particle trace visualization of the aortic specimens confirmed (**a**) near-physiological flow before surgery (first column, native aorta). (**b**) After aortotomy (sham surgery), a secondary flow pattern (asterisk) develops at the level of the surgical incision in the ascending aorta. (**c**) More secondary flow patterns developed after aortic valve replacement. Some patterns cannot be detected in this view but only after rotation of the aorta as shown in (**d**)

After sham surgery one small to medium-sized secondary flow pattern developed in the ascending aorta at the site of the incision in all six specimens (Fig. 5, Table 1). AVR of any type caused quantitative and qualitative increases in secondary flow patterns. The most secondary flow patterns were observed distal to the BV (number of flow patterns = 3), followed by the AVneo (number of flow patterns = 2). BV2 with a subvalvular stenosis demonstrated a similar progression of secondary flow patterns from sham surgery to BV implantation compared to the normal BV1 specimen without subvalvular stenosis. MV was not associated with more secondary flow patterns than aortotomy alone. However, the single secondary flow pattern was more pronounced. The sinus vortices in the aortas with the native valve reached high into the ascending aorta. They were found in all aortas except for BV replacements where susceptibility artefacts from the metal frame may have obscured them.

**Table 1:** Count and grading of secondary flow patterns

	Count per aorta	Grading
NAV-A (*n* = 6)	1	Median I–II [IQR I–II]
AVneo (*n* = 2)	2	I, II, and II, III
BV (*n* = 2)	3	I, I, II, and I, II, III
MV (*n* = 2)	1	I, and III

Secondary flow patterns after NAV-A, AVneo, BV and MV replacement. Secondary flow patterns were graded on a Likert scale in a four-step approach [11]; also see Supplementary Material, Fig. S1.

IQR, interquartile range.

**Figure 6:**
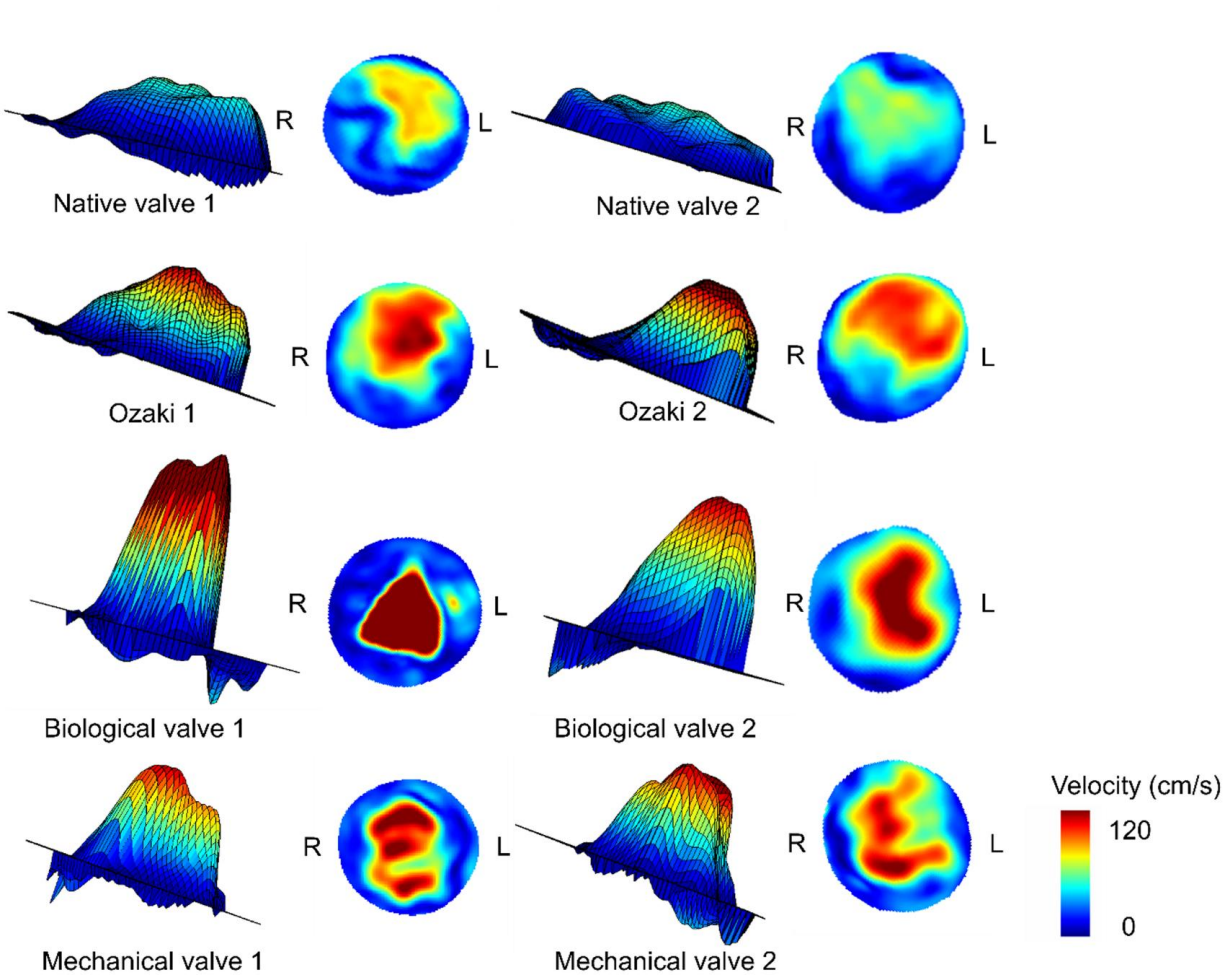
Mesh plots and velocity maps at the sinotubular junction show discrete differences of the flow profile between the native valve and different types of AVR. Orientation is marked with R (right) and L (left)

The model showed an unphysiological diastolic reflux due to setup constraints. Accordingly, we only evaluated systolic flow. Systolic stroke volume was 34 ± 3 ml. Implanting the *ex vivo* aorta into our setup was performed carefully but some leakage from tiny side branches could not be avoided. We estimate that 2–3 ml leaked into the box per cycle based on a ‘cardiac output’ measured with 4D flow MRI of approximately 2.2 l/min. Peak maximal velocity increased after all types of valve replacement compared to the native aorta (NAV = 75 ± 22 cm/s) and aortotomy (NAV-A = 79 ± 29 cm/s). We measured the highest peak maximal velocity distal to BV (142 ± 21 cm/s) followed by Ozaki (AVneo = 115 ± 36 cm/s) and MVs (107 ± 4 cm/s). After both BV replacements, a jet towards the outer curvature of the ascending aorta was found (Figs 5 and 6).

## DISCUSSION

We established an *ex vivo* model that allows for a comprehensive evaluation of individual surgical steps during different AVR strategies in a standardized setting. 4D flow MRI was successfully acquired for all specimens. Our results suggest that closure of a surgical incision of the aortic wall itself leads to changes in secondary flow pattern in the ascending aorta. Furthermore, all types of AVR led to alterations in systolic aortic flow pattern that were not present in the native specimens. We detected the most extensive secondary flow patterns distal to the BV followed by the Ozaki procedure and the MV.

Aortotomy and suture thereafter induced secondary flow patterns in all specimens. Our model enables us to test different incision and suturing techniques to identify the ones with the least impact on flow patterns in the short term. Long-time changes in aortic flow would need to be determined in a living animal model to allow for wound healing.

Flow patterns in the porcine aorta after BV replacement were comparable to flow patterns observed in patients with bicuspid aortic valve or aortic valve stenosis. These results are in line with previous studies in silicone models [4] and in patients [12, 13], where aberrant flow patterns and eccentric flow were found only after BV replacement but not after a MV. Several studies in patients with bicuspid aortic valves showed that valve-related, unphysiological aortic haemodynamics increase WSS [14, 15]. WSS measured with 4D flow MRI is significantly linked to macroscopic and microscopic alterations of the aortic wall: areas with increased WSS show thinning of elastic fibre in the aortic wall on histology. Aberrant flow and changes in WSS correlated with the incidence of aortic aneurysm growth in the ascending aorta in patients with bicuspid aortic valve [16, 17]. However, compared to severe aortic stenosis or regurgitation, flow disturbances after AVR are less severe and AVR seems to stop dilation of the aorta [18, 19]. It remains unclear if biological AVR influences aortic wall integrity in the long-term comparable to bicuspid valves.

In contrast, flow profiles after an Ozaki procedure and MV replacement were characterized by fewer flow alterations, which is in line with recent studies [12, 13]. 4D flow MRI in children after Ozaki or Ross procedures showed minor flow alterations flow after both procedures [20]. Haemodynamics in the ascending aorta of these children showed central flow (40% of Ross patients, 90% of Ozaki patients) or mild eccentricity. Unlike after BV replacement with a stented valve, a normal annular motion after the Ozaki procedure is preserved, allowing for more physiological haemodynamics [20, 21]. In addition, echocardiography of patients has proven a higher effective orifice area after the AVneo compared to BV replacement [22].

Comparison of the effective orifice area of the BV and MV did not differ significantly when evaluated in a mock circulation with a high-speed camera [23]. At lower stroke volumes, comparable to our experiment, effective orifice areas of BV and MV were also comparable to the native aortic valve as well as Ozaki neocuspidization. Furthermore, cusp motion throughout the cardiac cycle is similar between AVneo and the native valve, while this was significantly altered in stented BV [23]. AVneo and BV techniques use pericardium as valve cusps—either the patient’s own (Ozaki [1]) or bovine pericardium (BV). However, the main difference is the frame of BVs to maintain their shape. This frame reduces annular motion [24], while in the Ozaki procedure, the pericardium is sewed directly into the native aorta. This could at least partly explain the increase in peak velocity and more disturbed flow patterns after BV replacement in contrast to a less disturbed flow distal to valve neocuspidization and MV replacement.

Clinical evaluation of mid- and long-term outcomes after BV and MV replacement have shown comparable long-term results for both valve types with more bleeding events for MV and more reoperations due to degeneration for BV [25–28]. The American [29] and European guidelines [30] on valvular heart disease recommend mechanical prostheses in patients younger than 50 and 60 years of age, mainly due to the better durability. It remains unclear if implantation of BV in younger patients and therefore a longer exposure to pathological aortic flow might negatively influence aortic wall and heart function. With increasing life-expectancy and a more frequent implantation of BV in younger patients, the impact of flow alterations may become apparent. Long-term follow-up studies that include 4D flow MRI to associate secondary flow patterns with outcome are warranted.

There are several limitations of this study. First, we only examined a small number of porcine aortas. However, this was an experimental study of *ex vivo* analysis that successfully established a new *ex vivo* model to comprehensively examine Ozaki, biological and mechanical AVR. Further studies should include a fast 4D flow MRI analysis of the non-operated model to confirm flow profiles prior to surgery. Furthermore, our *ex vivo* model only allowed analysis of systolic flow but did not allow analysis of diastolic flow patterns and valve competency. However, systolic flow patterns are the most important flow patterns regarding impact on the vessel wall and energy kinetics. In diastole, mainly the existence and grading of valve insufficiency is relevant which can be easily measured *in vivo*. Due to leakage from anastomoses with the post-mortem porcine tissue, it was not possible to increase the stroke volume in the current setup. A larger box to capture the leaked fluid could mitigate this issue in future studies.

As the porcine swine aortas were smaller than most human aortas, we used smaller prostheses and stroke volumes than typically seen in humans [31]. In most patients, the stroke volume is higher and the aortic annulus is wider allowing for larger prostheses. We assume that haemodynamics found in our experiment are scalable to human dimensions.

As this experimental model uses post-mortem porcine tissue, measurements can only be performed within a limited time frame. Flow evaluation after wound healing and the impact of scar tissue cannot be evaluated. Comprehensive AVR evaluation in an animal study with additional 4D flow MRI during and after wound healing would be the next step. The impact of this study on surgical recommendations in patients cannot yet be evaluated.

## CONCLUSION

We have successfully established a new *ex vivo* model to investigate aortic systolic flow patterns after different techniques of AVR in a controlled setting. Our data suggest that the incision in the ascending aorta to gain access to AVR induces alterations in aortic flow. All examined AVRs induced secondary flow patterns. The new Ozaki procedure induced more secondary flow patterns than MV but fewer than BV.

## Supplementary Material

ivaf087_Supplementary_Data

## Data Availability

The data underlying this article will be shared on reasonable request to the corresponding author.

## Notes

Presented at the Deutscher Röntgenkongress, Wiesbaden, Germany, April 30, 2022.

Presented at the Annual Meeting International Society for Magnetic Resonance in Medicine, London, UK, May 09, 2022.

Presented at the European Congress of Radiology, Vienna, Austria, July 15, 2022.

Presented at the Annual Meeting Radiological Society North America, Chicago, USA, December 01, 2022.

Presented at the Annual Meeting Deutsche Gesellschaft für Thorax-, Herz- und Gefäßchirurgie, Online, February 26, 2021.

## ABBREVIATIONS

AVR: Aortic valve replacement
BV: Biological valve
LVOT: Left ventricular outflow tract
MRI: Magnetic resonance imaging
MV: Mechanical valve
NAV: Native aortic valve
WSS: Wall shear stress
