# Supplementary material for: Comprehensive assessment of aortic flow before and after aortic valve replacement in an ex vivo porcine model with four-dimensional flow magnetic resonance imaging
Source: Interdiscip Cardiovasc Thorac Surg. 2025 Apr 9;40(4):ivaf087. doi: 10.1093/icvts/ivaf087 (PMC12022217; doi:10.1093/icvts/ivaf087)
Supplement: ivaf087_Supplementary_Data [file ivaf087_supplementary_data.zip › Supplemental_file_legend.docx]

**Supplemental file legend**

Supplementary figure 1: Secondary flow patterns are defined as flow deviating from main flow. They were graded on a Likert scale according to their diameter in relation to the vessel diameter as small (grade I, <1/3 vessel diameter), medium (grade II, 1/3-2/3 vessel diameter) and large (grade III, >2/3 vessel diameter). In this example, the biological valve was associated with a grade I and grade III pattern. The grade II pattern in the figure developed after aortotomy (sham surgery).
