# Supplementary figures and images for: Comprehensive assessment of aortic flow before and after aortic valve replacement in an ex vivo porcine model with four-dimensional flow magnetic resonance imaging
Source: Interdiscip Cardiovasc Thorac Surg. 2025 Apr 9;40(4):ivaf087. doi: 10.1093/icvts/ivaf087 (PMC12022217; doi:10.1093/icvts/ivaf087)

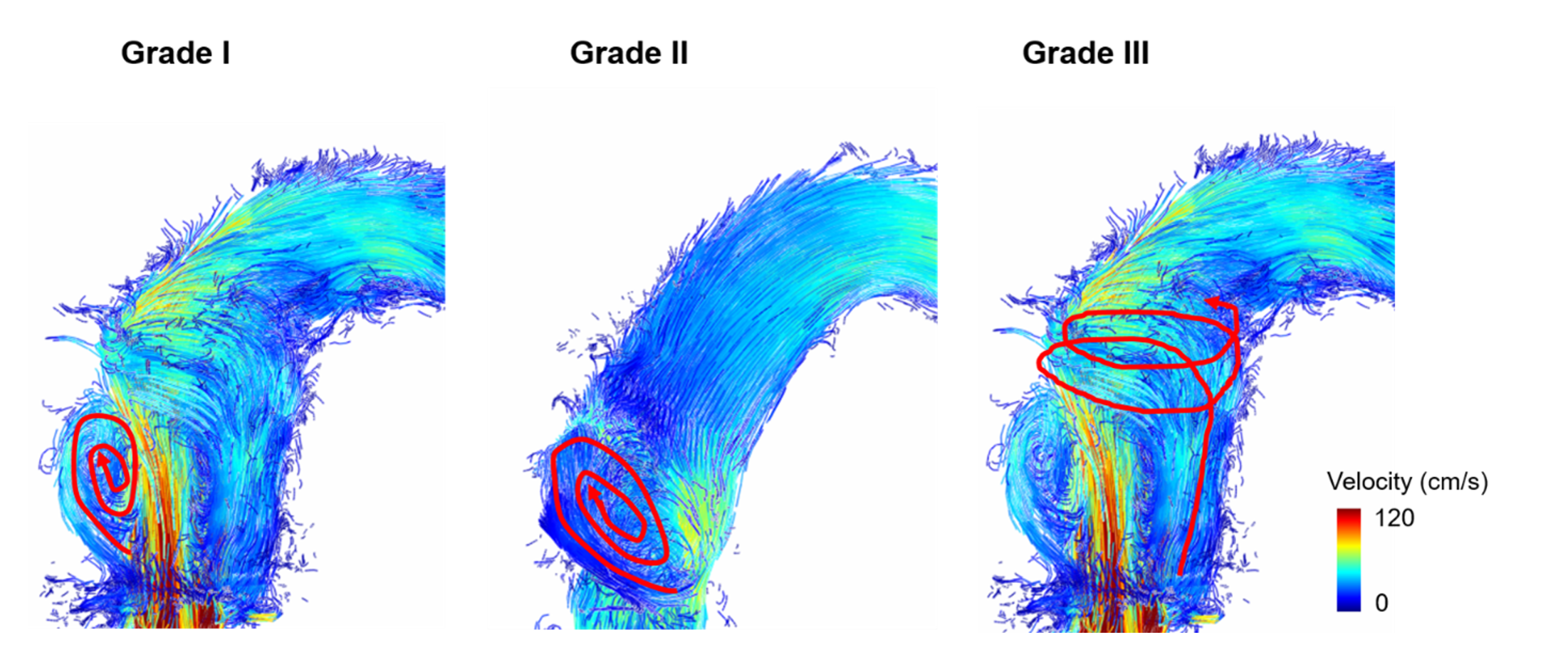

Supplement: ivaf087_Supplementary_Data [file ivaf087_supplementary_data.zip › Supplementary_figure_1.tif]
